# *Indian Journal of Psychiatry* and psychiatric research in India: Past, Present and Future

**DOI:** 10.4103/0019-5545.69197

**Published:** 2010-01

**Authors:** Ajai R. Singh

**Affiliations:** Editor, Mens Sana Monographs, Mumbai, India

**Keywords:** Indian Journal of Psychiatry, Psychiatric research in India, replication, refutation, self-correction, original research

## Abstract

Commendable work has been done in psychiatric research in India as it moves in tandem with contemporary trends abroad. Indian Journal of Psychiatry (*IJP*), as its flag-ship publication, has mirrored this trend faithfully down the decades. Stalwarts and icons of Indian psychiatry have set Indian research firmly on this course. A systematic appraisal of psychiatric research in India shows that most work is replicative, some of it corrective at the local level, and very little that is original and corrective at the international level. Opinion and policy makers, including *IJP* and research departments at colleges and universities, must endeavor to steer the course towards trend-setting and original work emanating from India, even as we do not neglect replicative work, of which we are masters.

## INTRODUCTION

There is much to commend in what has gone between the covers of the *Indian Journal of Psychiatry* as it enters its 52^nd^ year. More so, now, as it is indexed with PubMed; the cumulative effect of the work of editors and researchers down the decades, crystallizing in the work of, and propelling, the present editor and his board to work to this end. This indexing is a landmark in the history of Indian Psychiatry. A much desired[1] landmark that makes it so much more obvious that Indian Psychiatry, and *IJP*, must move forward as much to keep up with contemporary trends in international research, as to now go ahead and set some of them.

As far as the former is concerned – keeping up with international trends, Indian Psychiatry has done a lot of work, which is faithfully chronicled in the *IJP*. It would be a fruitful exercise for any interested researcher to scan archives of the journal available free access on its website[2] to see how research topics and trends abroad have been faithfully mirrored in the Journal, which has been the face of psychiatric research in the country down a major part of the latter half of the last century, as also the present. Right from its inception in 1958, if we scan its pages, we can see how well and faithfully, Indian Psychiatry has kept up with and echoed, research trends in Western psychiatry-whether it be work in psychodynamics, psychoanalysis,[3,4] insulin coma[5,6] and indigenous drugs[7] in the earlier decades; to behavior therapy,[8] prison psychiatry[9] and psychological tests[10,11] in the following; to diagnostics,[12] the philosophical,[13] phenomenological,[14] biological,[15] drug trials[16,17] [which continues even today],[18] social psychiatry,[19–21] psychosomatics,[22–24] cognitive behavior therapy,[25] and biology of psychiatry[26] in the last few decades; and to newer trends like terrorism,[27] internet addiction[28] and delusion,[29] evidence based medicine[30] and psychiatry,[31,32] standardized vignettes for research,[33] book reviews on research methodology,[34] clinical practice guidelines[35–37] in the last decade [References quoted here are but a representative sample].

Of course research and reviews of standard topics like schizophrenia research,[38] family in schizophrenia,[39] deficit schizophrenia,[40] fronto-temporal dysfunction[41] and disorders of aberrant neurodevelopment,[42] child mental health,[43,44] alcohol related problems,[45] psychotherapy in India,[46] ECT,[47] depression and cognition,[48] chronicity,[49] and nutrition[50] have occupied its pages. Studies on mania,[51] neuropsychology,[52] eroticism[53] sexual dysfunction[54,55] and women’s reproductive health[56] have also interested Indian researchers. This, again, is only a representative sample of the vast terrain Indian researchers have traversed down the decades, which only goes to underscore how faithfully they managed to mirror international psychiatric trends and concerns.

## TREND SET TOWARDS REMAINING CONTEMPORARY AND ADDRESSING LOCAL CONCERNS

It is indeed noteworthy that things have turned the way they have. Our trend setters and opinion makers, the stalwarts who not only headed psychiatric units in prestigious departments of psychiatry all over the country but also produced a vast quantum of research there from, set the course firmly in a direction that ensured we always remained contemporary with work at major research centers abroad, and never remained blinkered from international trends in research, even as we endeavored to study and adapt those findings to local concerns. Hence efforts to find Indian correlates of disorders, and test validity and reliability of psychological tests and diagnostic categories in Indian conditions remained, and still remains, a valid concern in psychiatric research in the country.[11,57] Moreover, democracy and openness to new trends from outside ensured that this occurred seamlessly here, so that the methods of scientific progress to which the West was exposed got its faithful echo in cultures like ours, which were in intimate scientific contact with them. This efficiently ensured that we were not left out of the great advance in psychiatry that the world witnessed in the decades during, and after, Freud’s and other masters’ influence in the field.

The efforts of Indian researchers to replicate western studies, to closely study research published there and reference them extensively, at times overbalancing by neglecting all but the most recent[58] as also research by their Indian counterparts,[59,60] were actually earnest attempts to establish linkage with contemporary world research and grow from there. In their quest to establish such linkages, they often failed to pause and take stock of where the whole enterprise was leading, firmly convinced that the direction burgeoning psychiatric research literature in the west was leading to was the direction to follow. Evaluative research and other writings about it, for example after 25 years[61] and 50 years[62] in the country, and after 25 years published in the journal,[63] futuristic writings about where we were headed,[64–66] the need and scope of international collaboration in psychiatric research,[67] what to do to increase international publication of research from developing nations,[68] psychopharmacology becoming a means to propagate commercial interests of industry,[69] with rejoinders to this position,[70,71] overdependence on pharmacotherapy,[72] ethics of publication,[73] spirituality and mental health,[74–76] prayer and healing,[77] and research tourism[1] have been raised, but rather few and far in-between. They need serious reflection on, and some forwarding, by those who have the interest of future Indian psychiatric research at heart.

## ANCIENT INDIA’S CONTRIBUTION TO PSYCHIATRY TODAY

A few studies on ancient India’s contribution to psychiatry were also proudly and reverentially presented, and, though a sprinkling in the whole research corpus, have made us feel satisfied that we did have much to say in our glorious past to which the present could take heed.[78,79] A few studies on yoga[80] and ayurvedic formulations[7] were also presented, which have no doubt aided this satisfaction. Studies on mind in Ayurveda and ancient India,[81,82] Bhagwad Gita,[83] guru-chela relationship,[84] Hanuman Complex[85] and the epistemology of mental phenomena[86] were courageous attempts to reflect some such pride, sometimes unknowingly. This was in step with trends in most other branches of knowledge in India, wherein the attempt was, on the one hand, to establish linkage with contemporary thought, largely western, but, on the other, also project that we had something significant to contribute, if not today, at least in the past, which could possibly have contemporary relevance.

This was only the reflection of a colonial mentality, which persists even today, though largely toned down, as psychiatric research in India finds its moorings. Looked at positively, they reflected nascent urges of a struggling culture trying to stand on its feet, and find a respectful self-identity. One could well understand their urges to remain contemporary so as not to be left out of recent developments, as well as retain a pride in the past which sustained a positive self image in the present. Therefore, reverential studies of our ancients continued to be proudly presented.

However, studies which critiqued our ancients’ contributions were conspicuous by their absence. For a culture seeking a fragile self-identity, it was possibly difficult, even dangerous. However, to make a significant contribution to science, one has to quickly progress from reverence to critical sifting, and a persistent and scorching critical scrutiny by the experimental method, and, also, the confirmation and/or refutation of peers. This still awaits doing in the present and the future. Till it is done, therefore, some will raise uncomfortable questions.[77,87] This unfinished item on the agenda need to be remedied on a war footing by those who feel ancient India had many concepts to give towards mental health in particular, and health in general.[88] Efforts to study the effects of prayer and healing,[77] as also questioning of the halo surrounding ancient thought,[87] though negative and rather caustic, are in a useful direction, as they attempt to further scientific enquiry in the phenomenon, and invite future researchers to refute their contentions with scientific proof.

### Editorials, presidential addresses and orations

Editorials, presidential addresses and orations published in the *IJP* down the decades have echoed contemporary concerns of their times – from research,[26] to prevention,[45] to the numerous psychiatric disorders,[43,45] to evidence based psychiatry,[31,32] to family in schizophrenia,[39] to psychiatry and women’s health,[56] to challenges of the times,[65] to revolutions,[89] and to making best use of what we have[90] etc.

While research papers are good indicators of what takes place in the research world in a certain area, editorials, presidential addresses and orations spell out the thinking of opinion makers and researchers who set the course and can bring about change. For those who want to study this, it would be an important exercise to closely study these items in the *IJP* archives,[2] for they are a rich repository of trends that guided the past and also set the course for now and the future. And those who believe they need to understand this course, and probably modify it, need to study it most closely.

It also makes obvious how editors, editorial boards, and their leanings decide what is published, and what research gets acceptance, which decides what research gets done. Presidential addresses are not just ego-trips or manifesto type window dressings. They often chart the course for the leader to follow, which percolates to the rank and file, and influences in which direction they proceed. Orations lay a recognized scholar’s lifetime work before an audience, and are therefore important indicators of such scholars’ work in the past and present, and what trends they wish to set for the future. Peers and juniors get overtly and covertly encouraged to follow such trends. That is how an association and its policies, including its research priorities and goals, progress.

This trend is worth delineation in systematic studies of editorials, presidential addresses and orations published in the *IJP*. This also is an unfinished agenda for the future.

## IN TUNE WITH, BUT A STEP BEHIND

All through these decades, Indian psychiatry has therefore firmly marched in tune with trends in the West, and felt amply justified it be so; marched in tune with, not in line with. Well, just a few steps behind, maybe, but always those steps behind; hardly ever in step with and hardly ever so as to set the tune for others to march to. And this is where some problem areas arise and need effective redressal.

I have desperately tried to scan the pages of the Journal for any enduring original concept or study which set the trend in international psychiatry that emanated from the pages of this journal and was unpleasantly surprised to find none.

None, in all the 52 years that we have had such a huge collection of research work published. And thereby hangs a tale.

The tale is of centuries of foreign rule that decimated original thinking in this land, and the confidence in its people that it could be produced here. This trend has shown reversal in only the last few years in Indian society, as it moves forward; and Indians, after opening up of their economy and globalization, are slowly getting over the awe of the western mind and systems. This confidence must no doubt percolate to all its endeavors, including research, and psychiatric research too. And lead to the conviction that original research in psychiatry is possible in India, which can set trends internationally.

A systematic reappraisal of trends and directions need to be deliberated over to bring about this change.

## SCIENTIFIC RESEARCH

There are essentially three classes of research- replicative, refutative, and self-correcting.

### Replication

Replicative research confirms another’s findings, and is the bread and butter of scientific research all over the world. It is very important, because it confirms an earlier researcher’s findings at another center and at a different time, and therefore confirms its acceptance, though always provisionally.

This brings about stability in science. In medicine, it is the basis for scientific research which finds clinical application, and therefore has the ability to promote human welfare.

### Refutation

The other type of research is refutative. This refutes the findings/theories of an earlier work or trend, and brings about change in thinking. If sufficiently large, and forceful, it can cause paradigmatic shifts and scientific revolutions. For example, the shifts from psychoanalysis to behavior therapy to biological approaches were such paradigmatic shifts which resulted from refutative studies.

This brings about progress in scientific work. It is the result of original path breaking and trend setting research, which others follow and replicate, till it is refuted. All innovations, new treatments and theories are the result of such work. When we shifted from custodial care to domiciliary care in schizophrenia, or from psychoanalysis to psychopharmacology in schizophrenia and depression, it was such a paradigm shift due to refutation that aided such a process.

To judge whether any work is trend-setting in this manner, a researcher must ask himself two fundamental questions:

1. What research/work/findings does the work in question refute, or falsify?
2. What research/work/findings will refute, or falsify, the findings of the present research/work/finding?

### Self-correction

There are also minimal self-corrections within systems, which result from partial replications and minimal refutations and also bring about scientific progress incrementally, almost covertly, a phenomenon which cannot be glossed over.

Such self-corrections bring about modifications in theory and therapy, without actually overturning any of them. Major corrections can take place this way all through science, and its greatest beneficiary in biomedicine. Psychiatry is no exception. When we progress from one SSRI to another, or even an SSRI to SNRI, it is often the result of such self corrections. When we progressed from typicals to atypicals in antipsychotics, it was because of such self-correction. It is change with stability, a golden mean between total change which destabilizes and total status quo which fossilises.

### Research in India largely replicative and locally self-corrective

Research in Indian psychiatry has been remarkable in being largely replicative.[91] Research done abroad is faithfully replicated in a few years here, with continuous reiteration of how our findings match those of their studies. Some, even as they report findings different from theirs, just report it. They never venture to comprehensively study and refute the findings of those researchers, except to say [and believe] that their findings were different probably due to local circumstances. While researchers who set trends abroad always try to prove the universality of their findings, and suggest there may be local variations, most Indian studies don’t have the nerve to say their findings, when refuting dominant trends, reflect a universal trend which may have local western variations.

This is where the crux of original research lies, if we have not only to promote research that is contemporary, but also set the agenda for present and future trends in international psychiatry.

For that, the essential attitudinal change that is needed is to believe that significant original research work, and theorizing, is possible in this country. To develop a theory consistently, and methodically, you need minds that engage in it, think they can do it well, and have the necessary expertise to do so. You also need the necessary research climate amongst peers and institutional heads that encourages such original work, and also submits it to critical scrutiny. Neglect by Indian authors of their own countrymen’s research is a legitimate lament.[59,60] But behind this lack of original research lies a serious self-doubt whether it is at all possible by Indians. This colonial mindset must first be changed by serious original work in all major fields of psychiatry.[92,93] This is not to say that we stop being aware of trends at other places. In fact we have to be very aware of what’s happening elsewhere. What we need to do is being aware, we stop only mouthing what others have said, and speak something of our own. To do so, we must first understand how the colonial mindset has tended to preoccupy minds even today,[94,95] and how some societies, notably the Australian, managed to overcome it.[96]

A significant step can also be set on the path of self-correction, which involves small but not insignificant incremental changes. Every researcher worth his salt in India, who has been in the field for at least a decade, knows the pitfalls of present research in his field, in India and abroad, and must not hesitate to point it out in a sustained and scientific manner. He often does so at the national level, but hesitates to do so at the international level; or may do so in private, but not in a scientific, systematic manner, which will be accepted by international peers. Personal and individual grudges and biases have no place in this process, although knowing human nature to be what it is, will no doubt creep in. That must be mercilessly eschewed, and as mercilessly sifted out by peers.

This trend towards refutation and self-correction must be set by the younger researchers. The senior researchers can also do it, if they can get rid of the colonial mind-set. If they cannot and we know how change is so difficult after a certain age and after establishing a certain reputation and they can at least realize in which direction the trend should now decisively change and encourage their juniors to do so.

### Trend towards original and internationally self-correcting research, while not neglecting the replicative

The trend must shift decisively towards original research, in whatever field of psychiatric research one specializes in; and academic and research excellence which spawns a future Nobel Laureate in psychiatry by 2020.[92] And of course, continue to replicate findings elsewhere in the world but to also refute them wherever necessary to take part in the processes of self-correction in one’s own thinking, and in the progress of psychiatry as a science, through the topic of one’s expertise.

There will be a necessary proliferation of ideas when we first do original work, many of them premature and weak. It’s like a newborn learning to walk, or a person with lifelong crutches doing so. A thorough and ruthless sifting and cleansing would be necessary before a work becomes accepted as an original, or even a corrected version of another’s original. Peers have to do this, but not in a manner that the nascent enterprise itself collapses. A new entrant in any field needs to be mentored, even as he is firmly guided on course by appreciation and constructively critical advice. What we may call critical mentoring.

This renaissance of Indian psychiatry need not be a distant goal. It is firmly achievable in the next few decades, if those engaged in research today, and their mentors and grant awarders, decide that is the course to chart. A lot depends on mentors and grant sanctioners everywhere. They often set the course for ’pragmatic’ researchers to follow, for researchers have to be pragmatic to survive, as also prosper; only let them not forget they have to be original to make enduring contributions.

### In closing

The contemporary Indian researcher in psychiatry must decide what course to set himself upon. As must his mentors, and the movers and shakers of Indian psychiatry. *IJP*, and its editorial policy, can be one such.

Of course, one only gets the government one deserves. So, if the rank and file of psychiatrists, and researchers in Indian psychiatry, feel this must indeed happen, the Journal will also faithfully mirror that trend. And the opinion makers in the field will sit up and take stock whether they feel this is the agenda to set, for the present and the future.

The vision is to not just survive, and be a receptor of knowledge, but also be its producer. To make signal and trend-setting advances in the field of psychiatric research. And not just in the country, but internationally. The stream of knowledge must flow as much from East to West as it flows from West to East today.

All well-wishers of Indian psychiatry and research must know what is the course to chart, for the present and the future. And any waylaying of this agenda, by rationalizations [that it is not possible because…] and/or denial [that it is not needed], must be firmly resisted.

## CONCLUSIONS

1. Indian psychiatric research has taken commendable steps to keep in tune with international trends.
2. *IJP*, as its face, has faithfully mirrored this down the decades.
3. Indian psychiatric research has been largely replicative. It has also at times been self-corrective at the Indian level, but hardly self-corrective or refutative/original at the international one. This is reflective of a colonial mindset whose remnants need urgent attention and repair.
4. Opinion makers in psychiatry, including the *IJP*, must set on the course to encourage more original and corrective work, even as it does not neglect replicative research. The younger researcher must take a conscious stand to follow this vision to bring about change.

## TAKE HOME MESSAGE

Indian Psychiatry has faithfully kept up with international trends. It is now time it sets some of them.
